# Evaluation of Repeated Web-Based Screening for Predicting Postpartum Depression: Prospective Cohort Study

**DOI:** 10.2196/26665

**Published:** 2021-12-10

**Authors:** Kathrin Haßdenteufel, Katrin Lingenfelder, Cornelia E Schwarze, Manuel Feisst, Katharina Brusniak, Lina Maria Matthies, Maren Goetz, Markus Wallwiener, Stephanie Wallwiener

**Affiliations:** 1 Department of Obstetrics and Gynecology Heidelberg University Heidelberg Germany; 2 Department of Psychology Heidelberg University Heidelberg Germany; 3 Institute of Medical Biometry Heidelberg University Heidelberg Germany

**Keywords:** postpartum depression, Edinburgh Postnatal Depression Scale, screening, pregnancy, algorithm

## Abstract

**Background:**

Postpartum depression (PPD) is a severe mental disorder that often results in poor maternal-infant attachment and negatively impacts infant development. Universal screening has recently been recommended to identify women at risk, but the optimal screening time during pregnancy has not been defined so far. Thus, web-based technologies with widespread use among women of childbearing age create new opportunities to detect pregnancies with a high risk for adverse mental health outcomes at an early stage.

**Objective:**

The aim of this study was to stratify the risk for PPD and to determine the optimal screening time during pregnancy by using a web-based screening tool collecting electronic patient-reported outcomes (ePROs) as the basis for a screening algorithm.

**Methods:**

In total, 214 women were repeatedly tested for depressive symptoms 5 times during and 3 times after pregnancy by using the Edinburgh Postnatal Depression Scale (EPDS), accessible on a web-based pregnancy platform, developed by the authors of this study. For each prenatal assessment, the area under the curve (AUC), sensitivity, specificity, and predictive values for PPD were calculated. Multivariate logistic regression analyses were applied to identify further potential predictors, such as age, education, parity, relationship quality, and anxiety, to increase predictive accuracy.

**Results:**

Digitally collected data from 214 pregnant women were analyzed. The predictive accuracy of depressive symptoms 3 and 6 months postpartum was reasonable to good regarding the screening in the second (AUC=0.85) and third (AUC=0.75) trimester. The multivariate logistic regression analyses resulted in an excellent AUC of 0.93 at 3 months and a good AUC of 0.87 at 6 months postpartum.

**Conclusions:**

The best predictive accuracy for PPD has been shown for screening between the 24th and the 28th gestational week (GW) and seems to be beneficial for identifying women at risk. In combination with the aforementioned predictive factors, the discriminatory power improved, particularly at 3 months postpartum. Screening for depression during pregnancy, combined with the women’s personal risk profile, can be used as a starting point for developing a digital screening algorithm. Thereby, web-based assessment tools constitute feasible, efficient, and cost-effective approaches. Thus, they seem to be beneficial in detecting high-risk pregnancies in order to improve maternal and infant birth outcomes in the long term.

## Introduction

The perinatal period represents a period in life where women turn to digital resources, particularly in the field of medical health care [1]. The growing supply of e- and mHealth technologies and the increasing desire to access and monitor health data generate the need for an empirical proof as intervention and information tools [2-4].

Vice versa, the assessment of patient-reported outcomes (PROs) in pregnancy has been shown to be a highly valid method for data acquisition [5]. Characteristics of female m- and eHealth seekers during pregnancy encompass a younger age, a lower self-rated health status, being pregnant for the first time, and being easily influenced by online sources in terms of pregnancy [6]. Furthermore, current research has shown that especially women with a higher risk of depression and anxiety disorders use pregnancy apps more extensively [7].

According to the literature, especially pregnant women show increased vulnerability for the onset or relapse of a manifest depressive disorder during the perinatal period, as pregnancy and childbirth represent 2 major events in a woman’s life, along with substantial changes in their responsibilities [8-10]. The prevalence of postpartum depression (PPD) varies depending on study type, measurement, time of assessment, and nationality from 10% to 15% during the first year after childbirth [11-15]. According to the *International Statistical Classification of Diseases and Related Health Problems, Tenth Revision* (*ICD-10*), and the *Diagnostic and Statistical Manual of Mental Disorders, Fifth Edition* (*DSM-5*), diagnostic criteria, PPD is diagnosed if symptoms such as sadness, anhedonia, disturbance in appetite or sleep, fatigue, psychomotor symptoms, worthlessness/guilt, attention deficits, and suicidality persist for at least 2 weeks with a peripartum onset [16,17].

Regarding the first days postpartum, PPD must be distinguished from baby blues. While PPD occurs within the first year postpartum, baby blues affects 50%-85% of mothers during the first 10 days postpartum but usually ameliorates within 2 weeks [18]. Baby blues is characterized by transient mood swings, tearfulness, and mild depressive symptoms [19,20].

Prior research identified risk factors for PPD, such as a history of mental disorders, stressful life events, limited social support, low socioeconomic background, and especially depression and anxiety, that have been reported to be the strongest predictors for adverse mental health outcomes in the postpartum period [21-25].

Suffering from PPD not only constitutes a burden for the mothers and fam ilies but also has a high impact on early mother-child interaction and parenting [26,27]. The long-term effects for the child include impaired mother-child bonding [28] and cognitive, emotional, and behavioral problems [29,30].

However, PPD often remains undetected and thus untreated as women who suffer from depressive disorders are sometimes unable to evaluate their emotions and reluctant to seek support and help on their own [31,32]. Even though early identification and support for mothers at risk are crucial to prevent PPD, only 20% of affected women are detected in the perinatal period and around 10% of those women receive adequate treatment and support [10,33].

According to the current literature, the most commonly used screening tool for perinatal depression is the Edinburgh Postnatal Depression Scale (EPDS) [34]. This self-report questionnaire contains 10 items measuring depressive symptom severity during the past week and has been validated for antenatal and postnatal application [35]. In fast-paced clinical settings, it has been shown to be practicable, takes less than 5 minutes to complete, and is highly accepted by women with and without depression alike [36,37].

Current evidence suggests that there is an overall benefit of perinatal depression screening [38]. The American College of Obstetricians and Gynecologists (ACOG) recommends perinatal screening for depressive symptoms at least once during pregnancy and after childbirth [39]. However, no optimal screening time and routine have been established in obstetrical care so far. There is still uncertainty about which screening tool provides the best predictive accuracy and how often and at what time point it should be applied [40,41].

Thus, web-based pregnancy tools provide new opportunities for real-time data acquisition, including the feasibility to capture symptom deterioration and upcoming adverse events [6,42,43]. Growing mHealth technologies can be used for prevention and intervention of depressive disorders at an early stage and may even reduce barriers to seeking psychotherapeutic support [44-51]. The feasibility and acceptability of web-based depression screening by means of the EPDS have been shown in the previous literature. In comparison to conventional paper-based methods, patients perceived web-based technologies as more convenient, discrete, and favorable [52].

Only few studies have assessed whether PPD can be reliably predicted during pregnancy by using the EPDS. The overall results show high negative predictive values and specificity but low positive predictive values and sensitivity with a reasonable discriminatory power [53]. Although Lau et al [54] first described a strong correlation among depressive symptoms in the second trimester and up to 6 weeks postpartum, Meijer et al [53] and Venkatesh et al [55] assumed that predictive accuracy is limited but that it can be improved by adding the history of depression. Both recommend a cut-off threshold of >5 for initial screening, followed by clinical diagnostics if positive.

The aim of this study was to longitudinally monitor EPDS scores by monthly assessments during the second and third trimester of pregnancy as well as 1 week, 3 months, and 6 months postnatally. By using this longitudinal approach, this work aimed to find the best screening time during pregnancy, with the highest predictive accuracy for PPD, based on the use of a web-based pregnancy tracking tool. In the next step, we aimed to refine the discriminatory power by including the women’s personal risk profiles, such as prenatal maternal anxiety or depressive symptoms, age, education, parity, or relationship quality. The absence of a standardized, widely accepted screening program in routine prenatal care underlines the need for testing the feasibility and validity of web-based data acquisition methods.

## Methods

### Participants and Study Design

**Figure 1:**
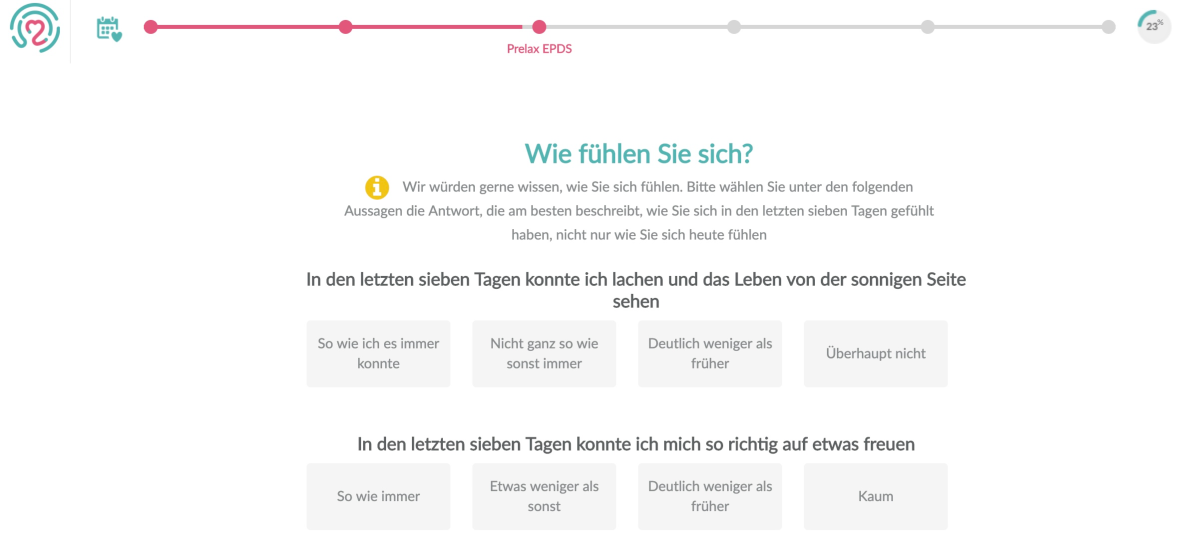
Screenshot of the PiiA platform. PiiA: *Patient-informiert-interaktiv-Arzt*.

This prospective bicentric study based on electronic patient-reported outcome (ePRO) questionnaires was conducted between October 2016 and September 2018 in the maternity departments of the University Hospitals Heidelberg and Tuebingen, Germany. In total, 214 pregnant women participated in the study. Inclusion criteria for recruitment were maternal age >18 years, sufficient knowledge of the German language, adequate internet access, and a singleton pregnancy between the 20th and the 27th gestational week (GW). Exclusion criteria encompassed a multiple pregnancy and known fetal anomalies and malformations. After participants provided their written consent to participate in the study, they completed the first set of web-based questionnaires on a tablet device on-site after registration with pseudo-anonymous user credentials on a self-developed platform called *Patient-informiert-interaktiv-Arzt* (patient-informs-interactively-physician [PiiA]; see Figure 1). At the time of enrollment, trained clinical staff provided participants assistance. Furthermore, an online tutorial was provided on the platform explaining the technical use. Further data acquisition was supposed to take place in the participants’ domestic environment on their preferred device. The participants received web-based assessments at 8 time points: every 4 weeks during pregnancy (Prae1=20th, Prae2=24th, Prae3=28th, Prae4=32nd, and Prae5=36th GW) up to 6 months postpartum (Post1=7 days, Post2=3 months, and Post3=6 months postpartum). In addition, 2 days prior to the scheduled assessment as well as 3 and 5 days afterward, the participants received an email reminder from the study team to complete the web-based questionnaires. Furthermore, sociodemographic and health-related data were obtained by means of ePROs.

To monitor maternal symptoms of depression and anxiety during the entire antenatal and postnatal period, the EPDS was applied at every time point, whereas the State-Trait Anxiety Inventory (STAI) was applied twice, at the 24th and the 32nd GW. The participants’ data were stored pseudo-anonymized and securely on a local storage device.

### Ethics

Ethics approval was obtained from the ethics committee at Heidelberg University (project no. S158/2016).

### Instruments

#### Edinburgh Postnatal Depression Scale

The EPDS [34] is a widely used screening tool validated for assessing pre- and postpartum symptoms of depression [35,56]. The 10-item self-report questionnaire measures depressive symptoms during the past 7 days. Every question is scored from 0 (no depressive symptoms) to 3 (severe depressive symptoms); thus, the total score varies between 0 and 30. Higher scores indicate a higher risk of minor or major depression. The recommended cut-off score is ≥10 points, which predicts minor depression and has shown good sensitivity and specificity of 0.96 and 1, respectively [57].

#### State-Trait Anxiety Inventory

The STAI [58] is used to assess anxiety as a temporary condition (State-Trait Anxiety Inventory, state scale [STAI-S]) as well as general anxiety as a personal trait (State-Trait Anxiety Inventory, trait scale [STAI-T]). In this study, the German versions of the STAI-T as well as the STAI-S were administered [59]. The questionnaire contains 20 items measured on a 4-point Likert scale from 1 (low) to 4 (high). Total scores vary from 20 to 80. In previous studies, the STAI has shown good discriminatory and predictive validity in perinatal populations [60]. The recommended cut-off score of >40 reached a sensitivity of 80.95%, a specificity of 79.75%, and a positive predictive value of 51.5% [61].

#### Questionnaire on Relationship Quality

The questionnaire on relationship quality (*Partnerschaftsfragebogen* [PFB]) [62,63] was applied to assess the participants’ self-rated relationship quality and satisfaction with their partners. The questionnaire encompasses 3 scales: (1) tenderness, (2) communication, and (3) conflict behavior. Each subscale contains 10 items measured on a 4-point Likert scale. While higher scores on the conflict behavior scale indicate less satisfaction, higher scores on the tenderness and communication scales indicate a higher relationship quality. In a representative study conducted on a German population, good to very good reliability was achieved for all 3 subscales (conflict behavior: α=.88; tenderness: α=.91; communication: α=.85; total scale: α=.93) [64].

### Statistical Analyses

Descriptive statistics, including mean scores, absolute and relative frequencies, and standard deviations, were used to analyze demographic variables of the entire study sample. The prevalence of antenatal and postnatal depressive symptoms (EPDS ≥ 10) was calculated at each point of assessment. To evaluate the discriminatory power of the EPDS, we constructed receiver-operating characteristic (ROC) curves for each of the relevant assessment times (5 antenatal assessments vs 3 postnatal assessments) by plotting sensitivity against 1-specificity for all possible cut-off values. Afterward, the respective area under the curve (AUC) was calculated for each ROC curve. A high AUC indicates a good selection of women with PPD symptoms as distinguished from those without. A widely used classification of the AUC is as follows: ≤0.50: useless (worse than a coin flip); 0.50-0.70: poor; 0.70-0.80: reasonable; 0.80-0.90: good; and >0.90: excellent [65]. Moreover, specificity, sensitivity, and positive and negative predictive values for specific cut-offs of the EPDS were calculated.

The predictive value of the EPDS was investigated by using multivariate logistic regression models. Here, the models were adjusted for the following factors [21-23]: age (in years), EPDS score at the best predictive time point of assessment during pregnancy, educational level, parity (previous births), general anxiety (STAI), and partnership quality (PFB). The models were described by odds ratios (ORs, 95% CI), *P* values, and the AUC compared to the AUC based on the respective best EPDS score.

*P* values were set at <.05. All analyses were performed using the statistics software R [66] based on the packages pROC [67] and PRROC [68].

## Results

### Sociodemographic and Birth-Specific Characteristics

In total, 214 women aged 22-44 years (mean age 33.5 years) completed the self-report questionnaires provided on the web-based pregnancy platform PiiA and were included in the analyses. The majority of the participants were married or in a relationship and well educated with a higher socioeconomic status. More than half of the women already had at least 1 child and were working part-time during pregnancy. About 14% of the children were born prematurely, and 48.6% were delivered vaginally without complications. Table 1 shows the sociodemographic data and further sample characteristics in detail.

**Table 1:** Distribution of sociodemographic and birth-specific variables (N=214).

Characteristic		Mean, n (%)
**Relationship status**		
	Married	154 (72.0)
	In a relationship	52 (24.3)
	Without a partner	5 (2.3)
	Missing data	3 (1.4)
**Parity**		
	0	85 (39.7)
	1+	124 (57.9)
	Missing data	5 (2.3)
**Graduation**		
	No degree	7 (3.3)
	General or intermediate degree	74 (34.6)
	Advanced technical certificate	25 (11.7)
	A level	101 (47.2)
	Other	4 (1.9)
	Missing data	3 (1.4)
**Employment of participants at time of recruitment**		
	Full-time	3 (1.4)
	Part-time	171 (79.9)
	Unemployed	29 (13.6)
	Missing data	11 (5.1)
**Monthly family income**		
	<€1000 (<US $1130.01)	20 (9.4)
	€1000-€2000 (US $1130.01-$2260.03)	60 (28.0)
	€2000-€3000 (US $2260.03-$3390.04)	41 (19.2)
	>€3000 (>US $3390.04)	84 (39.3)
	Missing data	9 (4.2)
**GW^a^ of delivery**		
	<38th GW	30 (14.0)
	≥38th GW	162 (75.7)
	Missing data	22 (10.3)
**Mode of delivery**		
	Vaginal delivery	104 (48.6)
	Planned cesarean section	34 (15.9)
	Unplanned cesarean section	47 (22.0)
	Forceps delivery or vacuum extraction	18 (8.4)
	Missing data	11 (5.1)

^a^GW: gestational week.

### Prevalence of Depressive Symptoms Pre- and Postnatally

During pregnancy, a reasonable number of women in our study sample were at risk for a depressive episode (EPDS ≥ 10): 12.6% (22/175) at the 20th GW, 25% (48/192) at the 24th GW, 18.2% (36/198) at the 28th GW, 13.2% (27/205) at the 32nd GW, and 18.3% (33/180) at the 36th GW. After childbirth, 18.5% (34/184) of the women showed an increased risk for PPD 7 days postpartum, 9.6% (13/136) at 3 months postpartum, and 13.2% (18/136) at 6 months postpartum.

### Predictive Value of PPD Symptoms

Table 2 presents the results of ROC analysis. The best predictive values according to the risk of PPD symptoms *7 days postpartum* (Post1) are shown in the 32nd GW (Prae4). The AUC was reasonable (0.76), and the specificity and negative predictive value for a cut-off of 10 were high at 93.0% and 87.1%, respectively, while the sensitivity and positive predictive values were moderate at 60.8% and 85.1%, respectively. Regarding depressive symptoms *3 months postpartum* (Post2), the AUC had good predictive accuracies in the 24th (Prae2; AUC=0.85) and 28th (Prae3; AUC=0.84) GW, showing once again high specificity (80.4% vs 84.5%) and negative predictive values (98.0% vs 96.0%) and lower sensitivity (83.3% vs 64.3%) and positive predictive values (27.0% vs 36.0%).Regarding the last measurement *6 months postpartum* (Post3), the best predictive accuracy was found in the 28th GW (Prae3), with a reasonable AUC of 0.75, high specificity (84.5%), high negative predictive value (93.9%), moderate sensitivity of 60.0% and low positive predictive value of 34.0%.

**Figure 2:**
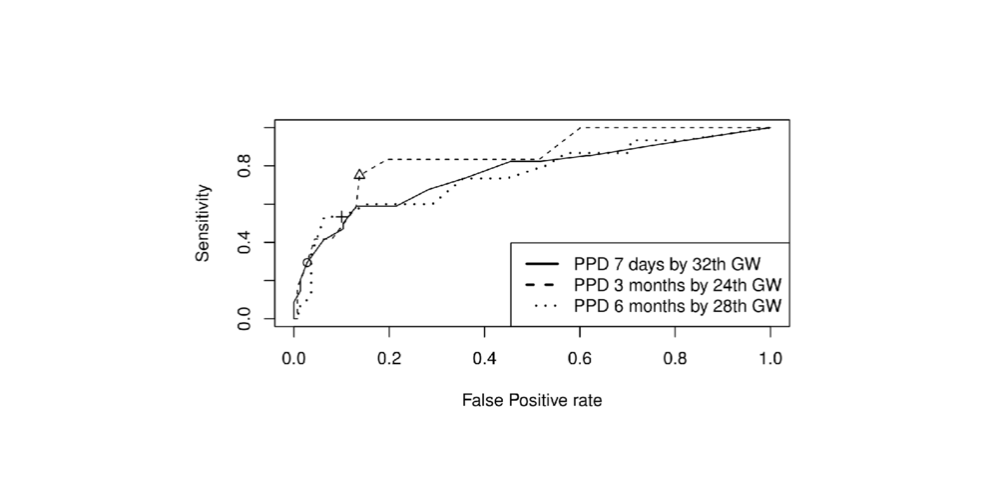
Highest AUC values for each postnatal assessment and the respective prenatal assessment including sensitivity and specificity. AUC: area under the curve; circle, cross, and triangle: respective optimal cut-off points (according to the Youden index); GW: gestational week; PPD: postpartum depression (at 7 days, 3 months, and 6 months postpartum).

The highest AUC values for the respective postnatal assessments are presented in Figure 2. The dots indicate the EPDS cut-off value of 10.

**Table 2:** Area under the curve for prediction of postpartum depression (EPDS^a^≥10) by means of depressive symptoms assessed during pregnancy.

PPD^b^	AUC^c^ at 20th GW^d^	AUC at 24th GW	AUC at 28th GW	AUC at 32nd GW	AUC at 36th GW
7 days	0.73	0.69	0.64	*0.76* ^e^	0.76
3 months	0.79	*0.85* ^e^	*0.84* ^e^	0.74	0.79
6 months	0.70	0.74	*0.75* ^e^	0.72	0.70

^a^EPDS: Edinburgh Postnatal Depression Scale.

^b^PPD: postpartum depression.

^c^AUC: area under the curve.

^d^GW: gestational week.

^e^All values in italics are significant.

### Detecting Predictors for Postnatal Depression

Multivariate logistic regression analyses revealed that postnatal symptoms of depression (assessed at 7 days postpartum) were significantly predicted by the depression scores during pregnancy (assessed at the 32nd GW, *P*<.001). Depressive symptoms 3 months postpartum were also predicted by prenatal depressive symptoms (assessed at the 24th GW, *P*=.02). Furthermore, the factor parity showed a significant influence (*P*=.049).

In the regression model regarding the time point at 6 months postpartum, the depression score during pregnancy at the 28th GW showed a highly significant impact (*P*=.001). The variable trait anxiety (STAI-T), assessed at 24 and 28 weeks prenatally, showed a tendency (*P*=.12) to increase but did not become statistically significant. Furthermore, in the multivariate models for PPD, the AUCs could be even improved by including the aforementioned factors compared to the univariate models. At 3 months postpartum, an excellent AUC of 0.93 in the multivariate model versus 0.85 in the univariate model was found. At 6 months postpartum, we found an AUC of 0.87 in the multivariate model versus 0.75 in the univariate model. Hence, the results of the multivariate logistic regression models show the best predictive accuracy for PPD for screening between the 24th and the 28 GW and are displayed in Table 3.

**Table 3:** Uni- and multivariate logistic regression models for the potential confounders age, prenatal depression, state and trait anxiety, education, parity, and partnership quality at 1 week, 3 months, and 6 months postpartum.

Variable	PPD^a^ 7 days		PPD 3 months		PPD 6 months	
	OR^b^ (95% CI)	*P* value	OR (95% CI)	*P* value	OR (95% CI)	*P* value
Age	0.97 (0.87-1.08)	.62	1.16 (0.96-1.44)	.14	1.02 (0.87-1.20)	.80
EPDS^c^	1.41 (1.23-1.65)	*<.001* ^d^	1.38 (1.12-1.83)	*.01* ^d^	1.24 (1.09-1.43)	*.001* ^d^
Education	0.69 (0.25-1.87)	.47	1.30 (0.22-9.05)	.77	1.179 (0.253-5.728)	.83
Parity	1.318 (0.451-4.032)	.62	0.118 (0.002-.855)	*.05* ^d^	1.043 (0.194-5.868)	.96
STAI-S^e^	1.007 (0.949-1.067)	.82	1.064 (0.922-1.132)	.69	0.998 (0.906-1.08)	.96
STAI-T^f^	0.953 (0.895-1.012)	.12	1.064 (0.951-1.213)	.31	1.083 (0.985-1.211)	.12
PFB^g^	0.500 (0.163-1.509)	.22	1.967 (0.303-16.477)	.5	1.348 (0.230-8.699)	.74
Multivariate AUC^h^	0.82	—^i^	*0.93* ^d^	—	0.87	—
Univariate AUC	0.76	—	0.85	—	0.75	—

^a^PPD: postpartum depression.

^b^OR: odds ratio.

^c^EPDS: Edinburgh Postnatal Depression Scale.

^d^All values in italics are significant.

^e^STAI-S: State-Trait Anxiety Inventory, state scale.

^f^STAI-T: State-Trait Anxiety Inventory, trait scale.

^g^PFB: *Partnerschaftsfragebogen*.

^h^AUC: area under the curve.

^i^Not applicable.

## Discussion

### Principal Findings

Due to the high prevalence of PPD and the high percentage of women who remain undiagnosed and untreated, this study aimed to define the optimal time point for web-based self-report screening for women at risk of PPD during pregnancy. We increased the impact of our results by conducting a prospective longitudinal study with a detailed assessment of maternal symptoms of depression at 8 follow-up assessments from the 20th GW onward up to 6 months postpartum, accessible on the self-developed web-based platform PiiA. We could also prove that the inclusion of further personal factors can significantly improve the accuracy of predicting PPD symptoms, which can potentially be used as a starting point for developing a screening algorithm.

### Implementation of an Online-Based Pregnancy-Screening Tool Using ePROs

The antenatal period represents a window of opportunity for adapting a healthy lifestyle, on the one hand, and to successfully integrate technology in clinical care, on the other hand.

Thus, the implementation of a web-based screening algorithm to detect women at risk should be a common aim for the health benefit of both expecting mothers and their offspring [38]. Previous research has already shown that electronic data acquisition represents equal validity and reliability compared to paper-based methods [69,70]. Furthermore, a personalized lifestyle intervention or psychoeducational treatment approach in order to improve health-related behavior seems to be beneficial, especially among women with mild-to-moderate symptoms of depression or anxiety [71]. Former studies have reported higher compliance rates, optimized cost efficiency, and better overall mental health outcomes due to web-based therapeutic tools compared to traditional practices [72]. Even a short-term electronic mindfulness-based intervention program resulted in reduced anxiety levels in pregnant woman after just 1 week of use [73].

However, due to the rising amount of easily accessible web-based sources, there are also existing tools without scientific evaluation and a considerable risk of misinformation [74,75]. Only an estimated marginal amount of 6% of the available health-related apps are validated in a scientific manner [76]. Thus, due to the evolving digitalization in medicine, there is an emerging need to test web-based tools for reliability and internal consistency before integrating into standard clinical practice. However, the presented results prove that web-based screening for detecting early symptoms of depression and anxiety by means of the EPDS is suitable. In a further approach, the EPDS should be implemented in web-based format at best into routine clinical obstetrical care. Hence, this could be the first step toward the establishment of a standardized screening algorithm for adverse mental health outcomes in the peripartum period.

### Prevalence of Depressive Symptoms During the Course of Pregnancy Until 6 Months Postpartum

In this prospective cohort study, we found that a high percentage of women who developed PPD symptoms already showed significantly higher antenatal EPDS scores. These findings are in line with previous studies showing that depressive symptoms may be more common during than after pregnancy [77,78]. Regarding merely the period of pregnancy, the highest prevalence rates were found at the 24th GW, initially followed by a decrease and then an increase at the end of pregnancy. These findings are comparable to the prevalence rates reported by Bennet et al [79]. Using an exploratory approach, Lau et al [54] (2010), who observed the same phenomenon, suggested that the relatively high prevalence of depressive symptoms during the second trimester might be caused by a high percentage of affected women who are undiagnosed and untreated [80,81]. Likewise, the possible persistence of pregnancy-related physical adverse events that can negatively affect mental health and the discontinuation of antidepressant medication that may lead to recurrent depression are conceivable [81,82].

Consistent with previous studies, we found that the proportion of women with depressive symptoms in the postpartum period (34/184, 18.5%) was similar to that in the third trimester (33/180, 18.3%). The increasing number of women who show depressive symptoms in the third trimester of pregnancy might be explained by greater physical discomfort, increased anxiety about the upcoming childbirth, and transition into a new role as a mother [83]. After delivery, the high prevalence of depressive symptoms can be explained by the onset of baby blues shortly after childbirth [84]. Baby blues being a transient condition, the prevalence of depressive symptoms in our sample decreased 3 months postpartum to 9.8% and increased slightly once again 6 months postpartum to 13.2%. These findings are in line with the results of a review published in 2018, in which an increase in depressive symptoms 6 months postpartum was reported and may reflect the added stress due to caring for a newborn and the constant demands of the infant [85]. A similar prevalence of PPD measured by the EPDS (cut-off > 10) was found in another German sample: 20.4% at 2 weeks postpartum, 15.8% at 6 weeks postpartum, and 15.4% 3-5 months postpartum [13,86].

### Predictive Accuracy of PPD Symptoms

The predictive accuracy for PPD 1 week postpartum increases with the progress of pregnancy, with the best results at the 32nd and the 36th GW. These findings imply that assessing depressive symptoms in late pregnancy gives a more precise prediction of depressive symptoms after childbirth. An explanation of that finding might be the higher level of distress and anxiety toward childbirth and parenthood, which can continue up to a few days after childbirth, representing the baby blues [84]. Although baby blues is a rather transient condition, the prediction of PPD symptoms 3 and 6 months postpartum is of higher clinical relevance.

Referring to depressive symptoms 3 months postpartum, the antenatal depressive symptoms, assessed at the 24th and the 28th GW, showed good discriminatory power. Although the positive predictive value was low, with only 27%-36% of positively screened women, indeed, presenting depressive symptoms 3 months postpartum, the negative predictive value was remarkably high, between 96% and 98%. Thus, women with a negative screening between the 24th and the 28th GW are quite unlikely to develop PPD 3 months postpartum.

The assessment of antenatal depressive symptoms at the 28th GW showed the most promising results in predicting depressive symptoms 6 months postpartum. The test criteria are similar to 3 months postnatally with high specificity (84.5%) and negative predictive value (93.9%) and lower sensitivity (60%) and positive predictive value (34%).

In conclusion, our findings show that predictive accuracy is highest in the period between the 24th and the 28th GW among all other time points of assessment during pregnancy.

According to the overall discriminatory power and predictive accuracy of the antenatal EPDS scores, previous research has shown similar AUC values ranging from 0.66 to 0.78 [53-55]. All of these studies have in common a high specificity and negative predictive values but present lower sensitivities and positive predictive values. To increase the negative predictive value and not to miss a woman at risk, Meijer et al [53] and Venkatesh et al [55] recommend a lower cut-off of 5 for the initial screening. Thus, negative results would indicate a low risk for developing PPD, while positive results would require further observation. However, this may cause a high number of false-positive cases and require increased clinical effort [15,22,53].

Our findings suggest that antenatal screening using the EPDS between the 24th and the 28th GW is the best predictor for depressive symptoms 3-6 months postpartum. This time interval also showed the highest prevalence rates of antenatal depressive symptoms among our study cohort. This is in line with the study of Lau et al [54] confirming the strong relationship between the EPDS score in the second trimester and 6 weeks postpartum. In contrast to our findings, however, Meijer et al [53] assumed that the time point of screening, whether it is in the first, second, or third trimester, does not show any influence regarding the predictive accuracy.

### Further Potential Predictors for PPD Symptoms

Previous studies have shown that a better predictive power for PPD can be achieved by combining antenatal EPDS scores with other potential factors, such as partner support or a prior history of depression [22,55].

Our results show that prenatal symptoms of depression, measured by the EPDS, constitute strong independent predictors of PPD at all postnatal times of assessment. This finding confirms once again the strong association between ante- and postnatal depressive symptoms.

In addition, we detected parity as another marginally significant predictor for PPD 3 months postpartum. Regarding parity as a predictor for PPD, previous studies have reported heterogeneous findings. Giving birth for the first time is often considered a risk factor for PPD in the first month postpartum. One reason may be that women are confronted with totally changed living conditions and insecurities owing to their inexperience in parenting, while already experienced mothers are usually more familiar and have adapted to their new role [87-89]*.* Other studies, in contrast, have reported multiparity being a risk factor for PDD as caring for more than 1 child can lead to additional stress and may overwhelm mothers in their everyday life [78,90]. These heterogenous findings imply that the influence of parity cannot be generalized and that further research in this field is required.

Regarding the relationship between antenatal anxiety and postpartum depression, inconsistent findings have been reported in the current literature so far. Multiple studies have shown that perinatal anxiety and depression often occur simultaneously. In addition, early symptoms of depression, anxiety, and distress predict impending mental health problems during and after pregnancy [83,91]. Both trait as well as state anxiety have been shown to be significant and independent risk factors for PPD in previous research [53]. In the study of Grant et al [61], anxiety was more than sixfold increased among participants diagnosed with PPD (OR 6.12). In contrast, however, our findings presented no significant impact of anxiety on PPD and are thus in accordance with other studies. In the work of Austin et al [92], for instance, the STAI showed no significant association with PPD after controlling for antenatal depression scores on the EPDS. More recent prospective studies are in line with our results, confirming once again the heterogeneous impact of anxiety during pregnancy on postpartum mental health [93,94].

According to the previous literature, an older maternal age and a higher educational level are associated with a decreased risk of PPD [95,96]. However, we did not find any significant effects of age and education at any time of assessment. This may be due to the fact that our study group was at a marginally older age and consisted of well-educated women, predominantly. In our study group, the quality of partnership measured by the PFB did not show any significant impact as a predictor for PPD either. The majority of our participants live together with their partners and probably consider their social support as sufficient.

The discriminatory power improved significantly by including all these personal risk factors in the model, so the multivariate AUC resulted in excellent and good values for depressive symptoms 3 and 6 months postpartum.

Although the EPDS is a simple and universally applicable screening tool, every patient should be considered individually with a different and unique psychosocial risk profile. By paying attention to and screening for additional risk factors, more women at risk can be identified than by merely using the EPDS.

### Strengths and Limitations

One strength of our study is the prospective longitudinal and innovative design, including frequent and repeated measures of depressive symptoms (8 assessments: 5 prenatally and 3 postnatally), whereas previous studies have analyzed only 1 time point in the postpartum period.

All of the applied questionnaires are internationally established and were provided as online questionnaires. The online assessments were easily accessible, universally applicable, and cost efficient. Furthermore, the participants were able to complete the questionnaires comfortably in their home environment, which may have had positive effects on their compliance and may have reduced their barriers to reporting mental health symptoms and personal information about emotions, partnership, etc, due to the greater privacy and easy access allowed by using their own personal computer or smartphone.

Another strength of our study is that we assessed not only symptoms of depression in detail but also anxiety symptoms, which is often a comorbid condition [91,97]. Thus, we were able to control for confounding effects of anxiety. Additionally, we distinguished between state, trait, and pregnancy-related anxiety to capture a large variety of mental affections. Beyond this, we assessed a broad range of further possible confounding factors that might contribute to the prediction of PPD.

A limitation of our study is the well-known fact that the EPDS is a screening tool and does not generate a valid *DSM-5* diagnosis. Those women who scored ≥10 on the EPDS are only at higher risk for minor or major depression but still require established clinical diagnostic testing to confirm the presence of PDD. Furthermore, different cut-off thresholds are validated in studies and clinical screening. Regarding a cut-off of ≥10, the sensitivities range from 59% to 100% and the specificities range from 44% to 97% [98]. For antenatal use, however, lower cut-off values have to be considered [99]. Still, a universal and established cut-off point is lacking, which contributes to the difficult comparability among previous studies. However, the EPDS is capable of capturing even subclinical depressive symptoms, which also potentially impacts clinical outcomes [100].

As we could not distinguish between women who were depressive or nondepressive at baseline, there is also a risk of a self-selection bias, as mentally affected women are more likely to refuse a study participation [101]. Regarding the self-report data acquisition used in our study, there are diverging findings in previous research concerning accuracy and validity. Former research has claimed that the use of ePRO parameters can lead to response and recall bias and thus to a loss of validity, as affective symptoms are often overestimated [102,103]. Other studies, in contrast, are providing evidence of good reliability of self-administered questionnaires in clinical practice [104,105].

Our study group consisted of mostly well-educated women with a higher socioeconomic status, and nearly all are married or in a partner relationship. Therefore, these findings can only be generalized to a limited extent. Despite these limitations, our results provide valuable information of substantial clinical importance.

This is the first essential measure before creating a digital screening algorithm and implementing real-time PRO-derived data into clinical care in order to capture adverse mental and physical health symptoms as early as possible.

### Conclusion

Based on our results, we determined the best predictive accuracy of digital, self-report screening for PPD during pregnancy to be between the 24th and the 28th GW. Although the EPDS may not be sufficient for predicting PPD alone, and some new risk factors may contribute after childbirth, the predictive accuracy achieved an excellent value, especially in combination with the women’s personal factors, such as anxiety, age, education, parity, and partnership support. Systematic antenatal screening is important to identify the proportion of women at risk as early as possible and thus avoid the detrimental consequences of untreated depression for both mother and child. Therefore, increased awareness of affective disorders during and after pregnancy is needed. It is crucial to implement a valid online screening tool for symptoms of depression in clinical routine, and establishing a routine screening program between the 24th and the 28th GW might be most promising to identify both women at risk for depression during pregnancy and a high proportion of women at risk for PPD. Therefore, our results have the potential to be used as a starting point for developing a screening algorithm for perinatal depression. As the pregnancy period is an emerging target for health interventions, the clinical implementation of a tracking and screening tool regarding mental symptoms from the beginning of pregnancy seems to be applicable and beneficial. In the next stage of development, therapeutic and educational treatment modalities based on the upcoming possibilities due growing e- and mHealth technologies should be incorporated.

## Abbreviations

ACOG: American College of Obstetricians and Gynecologists
AUC: area under the curve
*DSM-5*: *Diagnostic and Statistical Manual of Mental Disorders, Fifth Edition*
EPDS: Edinburgh Postnatal Depression Scale
ePRO: electronic patient-reported outcome
GW: gestational week
*ICD-10*: *International Statistical Classification of Diseases and Related Health Problems, Tenth Revision*
OR: odds ratio
PFB: *Partnerschaftsfragebogen*
PiiA: *Patient-informiert-interaktiv-Arzt*
PPD: postpartum depression
ROC: receiver operating characteristic
STAI: State-Trait Anxiety Inventory
STAI-S: State-Trait Anxiety Inventory, state scale
STAI-T: State-Trait Anxiety Inventory, trait scale

## References

[1] Bert F, Gualano MR, Brusaferro S, De Vito E, de Waure C, Torre Gl, Manzoli L, Messina G, Todros T, Torregrossa MV, Siliquini R (2013). Pregnancy e-health: a multicenter Italian cross-sectional study on internet use and decision-making among pregnant women. J Epidemiol Community Health.

[2] Eysenbach G, Diepgen TL (2001). The role of e-health and consumer health informatics for evidence-based patient choice in the 21st century. Clin Dermatol.

[3] Lupton D, Pedersen S (2016). An Australian survey of women's use of pregnancy and parenting apps. Women Birth.

[4] Wellde PT, Miller LA (2016). There's an app for that!: new directions using social media in patient education and support. J Perinat Neonatal Nurs.

[5] Elliott JP, Desch C, Istwan NB, Rhea D, Collins AM, Stanziano GJ (2010). The reliability of patient-reported pregnancy outcome data. Popul Health Manag.

[6] Wallwiener S, Müller M, Doster A, Laserer W, Reck C, Pauluschke-Fröhlich J, Brucker SY, Wallwiener CW, Wallwiener M (2016). Pregnancy eHealth and mHealth: user proportions and characteristics of pregnant women using web-based information sources-a cross-sectional study. Arch Gynecol Obstet.

[7] Mo Y, Gong W, Wang J, Sheng X, Xu DR (2018). The association between the use of antenatal care smartphone apps in pregnant women and antenatal depression: cross-sectional study. JMIR Mhealth Uhealth.

[8] Goodman JH, Guarino A, Chenausky K, Klein L, Prager J, Petersen R, Forget A, Freeman M (2014). CALM pregnancy: results of a pilot study of mindfulness-based cognitive therapy for perinatal anxiety. Arch Womens Ment Health.

[9] Dunkel Schetter C, Tanner L (2012). Anxiety, depression and stress in pregnancy: implications for mothers, children, research, and practice. Curr Opin Psychiatry.

[10] Marcus SM (2009). Depression during pregnancy: rates, risks and consequences; Motherisk Update 2008. Can J Clin Pharmacol.

[11] Gavin NI, Gaynes BN, Lohr KN, Meltzer-Brody S, Gartlehner G, Swinson T (2005). Perinatal depression: a systematic review of prevalence and incidence. Obstet Gynecol.

[12] Woody CA, Ferrari AJ, Siskind DJ, Whiteford HA, Harris MG (2017). A systematic review and meta-regression of the prevalence and incidence of perinatal depression. J Affect Disord.

[13] Reck C, Struben K, Backenstrass M, Stefenelli U, Reinig K, Fuchs T, Sohn C, Mundt C (2008). Prevalence, onset and comorbidity of postpartum anxiety and depressive disorders. Acta Psychiatr Scand.

[14] Underwood L, Waldie K, D'Souza S, Peterson ER, Morton S (2016). A review of longitudinal studies on antenatal and postnatal depression. Arch Womens Ment Health.

[15] O'Hara MW, McCabe JE (2013). Postpartum depression: current status and future directions. Annu Rev Clin Psychol.

[16] Uher R, Payne JL, Pavlova B, Perlis RH (2014). Major depressive disorder in DSM-5: implications for clinical practice and research of changes from DSM-IV. Depress Anxiety.

[17] American Psychiatric Association Diagnostic and Statistical Manual of Mental Disorders, Fifth Edition.

[18] Matthey S (2008). Using the Edinburgh Postnatal Depression Scale to screen for anxiety disorders. Depress Anxiety.

[19] Henshaw C (2003). Mood disturbance in the early puerperium: a review. Arch Womens Ment Health.

[20] Beck CT (2006). Postpartum depression: it isn't just the blues. Am J Nurs.

[21] Beck CT (2001). Predictors of postpartum depression: an update. Nurs Res.

[22] Milgrom J, Gemmill AW, Bilszta JL, Hayes B, Barnett B, Brooks J, Ericksen J, Ellwood D, Buist A (2008). Antenatal risk factors for postnatal depression: a large prospective study. J Affect Disord.

[23] Hein A, Rauh C, Engel A, Häberle L, Dammer U, Voigt F, Fasching PA, Faschingbauer F, Burger P, Beckmann MW, Kornhuber J, Goecke TW (2014). Socioeconomic status and depression during and after pregnancy in the Franconian Maternal Health Evaluation Studies (FRAMES). Arch Gynecol Obstet.

[24] Bauer AE, Maegbaek ML, Liu X, Wray NR, Sullivan PF, Miller WC, Meltzer-Brody S, Munk-Olsen T (2018). Familiality of psychiatric disorders and risk of postpartum psychiatric episodes: a population-based cohort study. Am J Psychiatry.

[25] Haßdenteufel K, Feißt M, Brusniak K, Lingenfelder K, Matthies LM, Wallwiener M, Wallwiener S (2020). Reduction in physical activity significantly increases depression and anxiety in the perinatal period: a longitudinal study based on a self-report digital assessment tool. Arch Gynecol Obstet.

[26] Field T (2010). Postpartum depression effects on early interactions, parenting, and safety practices: a review. Infant Behav Dev.

[27] Milgrom J, Westley DT, Gemmill AW (2004). The mediating role of maternal responsiveness in some longer term effects of postnatal depression on infant development. Infant Behav Dev.

[28] Dubber S, Reck C, Müller M, Gawlik S (2015). Postpartum bonding: the role of perinatal depression, anxiety and maternal-fetal bonding during pregnancy. Arch Womens Ment Health.

[29] Goodman SH, Rouse MH, Connell AM, Broth MR, Hall CM, Heyward D (2011). Maternal depression and child psychopathology: a meta-analytic review. Clin Child Fam Psychol Rev.

[30] Netsi E, Pearson RM, Murray L, Cooper P, Craske MG, Stein A (2018). Association of persistent and severe postnatal depression with child outcomes. JAMA Psychiatry.

[31] Whitton A, Warner R, Appleby L (1996). The pathway to care in post-natal depression: women's attitudes to post-natal depression and its treatment. Br J Gen Pract.

[32] Dennis C, Chung-Lee L (2006). Postpartum depression help-seeking barriers and maternal treatment preferences: a qualitative systematic review. Birth.

[33] Marcus SM, Heringhausen JE (2009). Depression in childbearing women: when depression complicates pregnancy. Prim Care.

[34] Cox JL, Holden JM, Sagovsky R (1987). Detection of postnatal depression. Development of the 10-item Edinburgh Postnatal Depression Scale. Br J Psychiatry.

[35] Murray D, Cox JL (1990). Screening for depression during pregnancy with the Edinburgh Depression Scale (EDDS). J Reprod Infant Psychol.

[36] Brealey SD, Hewitt C, Green JM, Morrell J, Gilbody S (2010). Screening for postnatal depression: is it acceptable to women and healthcare professionals? A systematic review and meta‐synthesis. J Reprod Infant Psychol.

[37] Gemmill AW, Leigh B, Ericksen J, Milgrom J (2006). A survey of the clinical acceptability of screening for postnatal depression in depressed and non-depressed women. BMC Public Health.

[38] Reilly N, Kingston D, Loxton D, Talcevska K, Austin M (2020). A narrative review of studies addressing the clinical effectiveness of perinatal depression screening programs. Women Birth.

[39] American College of Obstetricians and Gynecologists (2015). Committee opinion no. 630: screening for perinatal depression. Obstet Gynecol.

[40] Knights JE, Salvatore ML, Simpkins G, Hunter K, Khandelwal M (2016). In search of best practice for postpartum depression screening: is once enough?. Eur J Obstet Gynecol Reprod Biol.

[41] Milgrom J, Gemmill AW (2014). Screening for perinatal depression. Best Pract Res Clin Obstet Gynaecol.

[42] Goetz M, Müller M, Matthies LM, Hansen J, Doster A, Szabo A, Pauluschke-Fröhlich J, Abele H, Sohn C, Wallwiener M, Wallwiener S (2017). Perceptions of patient engagement applications during pregnancy: a qualitative assessment of the patient's perspective. JMIR Mhealth Uhealth.

[43] Basch E, Deal AM, Kris MG, Scher HI, Hudis CA, Sabbatini P, Rogak L, Bennett AV, Dueck AC, Atkinson TM, Chou JF, Dulko D, Sit L, Barz A, Novotny P, Fruscione M, Sloan JA, Schrag D (2016). Symptom monitoring with patient-reported outcomes during routine cancer treatment: a randomized controlled trial. J Clin Oncol.

[44] Van Dijk MR, Huijgen NA, Willemsen SP, Laven JS, Steegers EA, Steegers-Theunissen RP (2016). Impact of an mHealth platform for pregnancy on nutrition and lifestyle of the reproductive population: a survey. JMIR Mhealth Uhealth.

[45] Rathbone AL, Prescott J (2017). The use of mobile apps and SMS messaging as physical and mental health interventions: systematic review. J Med Internet Res.

[46] van den Heuvel JF, Groenhof TK, Veerbeek JH, van Solinge WW, Lely AT, Franx A, Bekker MN (2018). eHealth as the next-generation perinatal care: an overview of the literature. J Med Internet Res.

[47] Firth J, Torous J, Nicholas J, Carney R, Pratap A, Rosenbaum S, Sarris J (2017). The efficacy of smartphone-based mental health interventions for depressive symptoms: a meta-analysis of randomized controlled trials. World Psychiatry.

[48] Lindhiem O, Bennett CB, Rosen D, Silk J (2015). Mobile technology boosts the effectiveness of psychotherapy and behavioral interventions: a meta-analysis. Behav Modif.

[49] Bowers K, Laughon SK, Kim S, Mumford SL, Brite J, Kiely M, Zhang C (2013). The association between a medical history of depression and gestational diabetes in a large multi-ethnic cohort in the United States. Paediatr Perinat Epidemiol.

[50] Hussain-Shamsy N, Shah A, Vigod SN, Zaheer J, Seto E (2020). Mobile health for perinatal depression and anxiety: scoping review. J Med Internet Res.

[51] Rebello TJ, Marques A, Gureje O, Pike KM (2014). Innovative strategies for closing the mental health treatment gap globally. Curr Opin Psychiatry.

[52] Kingston D, Austin M, Veldhuyzen van Zanten S, Harvalik P, Giallo R, McDonald SD, MacQueen G, Vermeyden L, Lasiuk G, Sword W, Biringer A (2017). Pregnant women's views on the feasibility and acceptability of web-based mental health e-screening versus paper-based screening: a randomized controlled trial. J Med Internet Res.

[53] Meijer JL, Beijers C, van Pampus MG, Verbeek T, Stolk RP, Milgrom J, Bockting CLH, Burger H (2014). Predictive accuracy of Edinburgh Postnatal Depression Scale assessment during pregnancy for the risk of developing postpartum depressive symptoms: a prospective cohort study. BJOG.

[54] Lau Y, Wong DFK, Chan KS (2010). The utility of screening for perinatal depression in the second trimester among Chinese: a three-wave prospective longitudinal study. Arch Womens Ment Health.

[55] Venkatesh KK, Kaimal AJ, Castro VM, Perlis RH (2017). Improving discrimination in antepartum depression screening using the Edinburgh Postnatal Depression Scale. J Affect Disord.

[56] Matthey S, Henshaw C, Elliott S, Barnett B (2006). Variability in use of cut-off scores and formats on the Edinburgh Postnatal Depression Scale: implications for clinical and research practice. Arch Womens Ment Health.

[57] Bergant AM, Nguyen T, Heim K, Ulmer H, Dapunt O (1998). German language version and validation of the Edinburgh Postnatal Depression Scale. Dtsch Med Wochenschr.

[58] Spielberger C, Gorsuch R, Lushene R (1970). Manual for the State-Trait Anxiety Inventory.

[59] Laux L, Glanzmann P, Schaffner P, Spielberger C (1981). Das State-Trait-Angstinventar: STAI; theoretische Grundlagen und Handanweisung.

[60] Meades R, Ayers S (2011). Anxiety measures validated in perinatal populations: a systematic review. J Affect Disord.

[61] Grant K, McMahon C, Austin M-P (2008). Maternal anxiety during the transition to parenthood: a prospective study. J Affect Disord.

[62] Hahlweg K (1996). Fragebogen zur Partnerschaftsdiagnostik (FPD).

[63] Hahlweg K (1996). Fragebogen zur Partnerschaftsdiagnostik (FPD).

[64] Hinz A, Stöbel-Richter Y, Brähler E (2001). Der *Partnerschaftsfragebogen*(PFB): Normierung und soziodemographische Einflussgrößen auf die Partnerschaftsqualität. Diagnostica.

[65] Hanley JA, McNeil BJ (1982). The meaning and use of the area under a receiver operating characteristic (ROC) curve. Radiology.

[66] R Core Team (2019). R: A Language and Environment for Statistical Computing.

[67] Robin X, Turck N, Hainard A, Tiberti N, Lisacek F, Sanchez J, Müller Markus (2011). pROC: an open-source package for R and S+ to analyze and compare ROC curves. BMC Bioinform.

[68] Grau J, Grosse I, Keilwagen J (2015). PRROC: computing and visualizing precision-recall and receiver operating characteristic curves in R. Bioinformatics.

[69] Wallwiener M, Matthies L, Simoes E, Keilmann L, Hartkopf AD, Sokolov AN, Walter CB, Sickenberger N, Wallwiener S, Feisst M, Gass P, Fasching PA, Lux MP, Wallwiener D, Taran F, Rom J, Schneeweiss A, Graf J, Brucker SY (2017). Reliability of an e-PRO tool of EORTC QLQ-C30 for measurement of health-related quality of life in patients with breast cancer: prospective randomized trial. J Med Internet Res.

[70] Matthies LM, Taran F, Keilmann L, Schneeweiss A, Simoes E, Hartkopf AD, Sokolov AN, Walter CB, Sickenberger N, Wallwiener S, Feisst M, Gass P, Lux MP, Schuetz F, Fasching PA, Sohn C, Brucker SY, Graf J, Wallwiener M (2019). An electronic patient-reported outcome tool for the FACT-B (Functional Assessment of Cancer Therapy-Breast) questionnaire for measuring the health-related quality of life in patients with breast cancer: reliability study. J Med Internet Res.

[71] Postpartum Depression: Action Towards CausesTreatment (PACT) Consortium (2015). Heterogeneity of postpartum depression: a latent class analysis. Lancet Psychiatry.

[72] Simon GE, Ludman EJ (2009). It's time for disruptive innovation in psychotherapy. Lancet.

[73] Goetz M, Schiele C, Müller M, Matthies LM, Deutsch TM, Spano C, Graf J, Zipfel S, Bauer A, Brucker SY, Wallwiener M, Wallwiener S (2020). Effects of a brief electronic mindfulness-based intervention on relieving prenatal depression and anxiety in hospitalized high-risk pregnant women: exploratory pilot study. J Med Internet Res.

[74] Connor K, Wambach K, Baird MB (2018). Descriptive, qualitative study of women who use mobile health applications to obtain perinatal health information. J Obstet Gynecol Neonatal Nurs.

[75] O'Donnell BE, Lewkowitz AK, Vargas JE, Zlatnik MG (2016). Examining pregnancy-specific smartphone applications: what are patients being told?. J Perinatol.

[76] Martínez-Pérez B, de la Torre-Díez I, López-Coronado M (2013). Mobile health applications for the most prevalent conditions by the World Health Organization: review and analysis. J Med Internet Res.

[77] Alder J, Fink N, Bitzer J, Hösli I, Holzgreve W (2007). Depression and anxiety during pregnancy: a risk factor for obstetric, fetal and neonatal outcome? A critical review of the literature. J Matern Fetal Neonatal Med.

[78] Banti S, Mauri M, Oppo A, Borri C, Rambelli C, Ramacciotti D, Montagnani MS, Camilleri V, Cortopassi S, Rucci P, Cassano GB (2011). Compr Psychiatry.

[79] Bennett HA, Einarson A, Taddio A, Koren G, Einarson TR (2004). Prevalence of depression during pregnancy: systematic review. Obstet Gynecol.

[80] Andersson L, Sundström-Poromaa I, Bixo M, Wulff M, Bondestam K, åStröm M (2003). Point prevalence of psychiatric disorders during the second trimester of pregnancy: a population-based study. Am J Obstet Gynecol.

[81] Cohen LS, Altshuler LL, Harlow BL, Nonacs R, Newport DJ, Viguera AC, Suri R, Burt VK, Hendrick V, Reminick AM, Loughead A, Vitonis AF, Stowe ZN (2006). Relapse of major depression during pregnancy in women who maintain or discontinue antidepressant treatment. JAMA.

[82] Mongini F, Keller R, Deregibus A, Raviola F, Mongini T, Sancarlo M (2003). Personality traits, depression and migraine in women: a longitudinal study. Cephalalgia.

[83] Rallis S, Skouteris H, McCabe M, Milgrom J (2014). A prospective examination of depression, anxiety and stress throughout pregnancy. Women Birth.

[84] Reck C, Stehle E, Reinig K, Mundt C (2009). Maternity blues as a predictor of DSM-IV depression and anxiety disorders in the first three months postpartum. J Affect Disord.

[85] Shorey S, Chee CYI, Ng ED, Chan YH, Tam WWS, Chong YS (2018). Prevalence and incidence of postpartum depression among healthy mothers: a systematic review and meta-analysis. J Psychiatr Res.

[86] Weidner K, Bittner A, Pirling S, Galle M, Junge-Hoffmeister J, Einsle F, Stöbel-Richter Y (2013). Was hält Schwangere gesund? Protektive Faktoren für postpartale Depression. Z Psychosom Med Psychother.

[87] Räisänen Sari, Lehto SM, Nielsen HS, Gissler M, Kramer MR, Heinonen S (2014). Risk factors for and perinatal outcomes of major depression during pregnancy: a population-based analysis during 2002-2010 in Finland. BMJ Open.

[88] Iwata H, Mori E, Sakajo A, Aoki K, Maehara K, Tamakoshi K (2016). Prevalence of postpartum depressive symptoms during the first 6 months postpartum: association with maternal age and parity. J Affect Disord.

[89] Glavin K, Smith L, Sørum R (2009). Prevalence of postpartum depression in two municipalities in Norway. Scand J Caring Sci.

[90] Sword W, Landy CK, Thabane L, Watt S, Krueger P, Farine D, Foster G (2011). Is mode of delivery associated with postpartum depression at 6 weeks: a prospective cohort study. BJOG.

[91] Skouteris H, Wertheim EH, Rallis S, Milgrom J, Paxton SJ (2009). Depression and anxiety through pregnancy and the early postpartum: an examination of prospective relationships. J Affect Disord.

[92] Austin M, Tully L, Parker G (2007). Examining the relationship between antenatal anxiety and postnatal depression. J Affect Disord.

[93] Cox JL, Connor YM, Henderson I, McGuire RJ, Kendell RE (1983). Prospective study of the psychiatric disorders of childbirth by self report questionnaire. J Affect Disord.

[94] Kumar R, Robson KM (1984). A prospective study of emotional disorders in childbearing women. Br J Psychiatry.

[95] Chien L, Tai C, Yeh M (2012). Domestic decision-making power, social support, and postpartum depression symptoms among immigrant and native women in Taiwan. Nurs Res.

[96] Abdollahi F, Sazlina S, Zain AM, Zarghami M, Asghari Jafarabadi M, Lye M (2014). Postpartum depression and psycho-socio-demographic predictors. Asia Pac Psychiatry.

[97] Masi G, Millepiedi S, Mucci M, Poli P, Bertini N, Milantoni L (2004). Generalized anxiety disorder in referred children and adolescents. J Am Acad Child Adolesc Psychiatry.

[98] Gibson J, McKenzie-McHarg K, Shakespeare J, Price J, Gray R (2009). A systematic review of studies validating the Edinburgh Postnatal Depression Scale in antepartum and postpartum women. Acta Psychiatr Scand.

[99] Kozinszky Z, Dudas RB (2015). Validation studies of the Edinburgh Postnatal Depression Scale for the antenatal period. J Affect Disord.

[100] Matthies LM, Wallwiener S, Müller M, Doster A, Plewniok K, Feller S, Sohn C, Wallwiener M, Reck C (2017). Maternal self-confidence during the first four months postpartum and its association with anxiety and early infant regulatory problems. Infant Behav Dev.

[101] van de Loo KFE, Vlenterie R, Nikkels SJ, Merkus PJFM, Roukema J, Verhaak CM, Roeleveld N, van Gelder MMHJ (2018). Depression and anxiety during pregnancy: the influence of maternal characteristics. Birth.

[102] Silva MMDJ, Nogueira DA, Clapis MJ, Leite EPRC (2017). Anxiety in pregnancy: prevalence and associated factors. Rev Esc Enferm USP.

[103] Evenson KR, Chasan-Taber L, Symons Downs D, Pearce EE (2012). Review of self-reported physical activity assessments for pregnancy: summary of the evidence for validity and reliability. Paediatr Perinat Epidemiol.

[104] Kominiarek MA, Balmert LC, Tolo H, Grobman W, Simon M (2019). A feasibility study of activity tracking devices in pregnancy. BMC Pregnancy Childbirth.

[105] Zimmerman M, Ruggero CJ, Chelminski I, Young D, Posternak MA, Friedman M, Boerescu D, Attiullah N (2006). Developing brief scales for use in clinical practice: the reliability and validity of single-item self-report measures of depression symptom severity, psychosocial impairment due to depression, and quality of life. J Clin Psychiatry.

